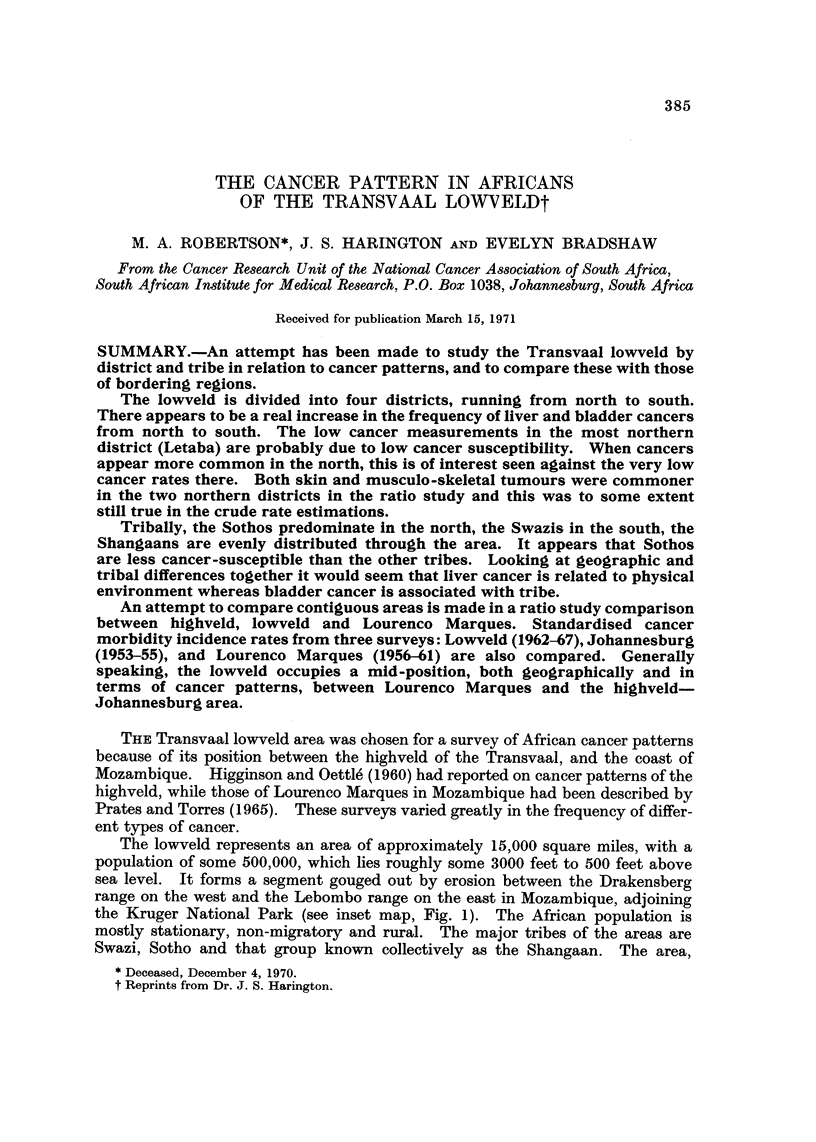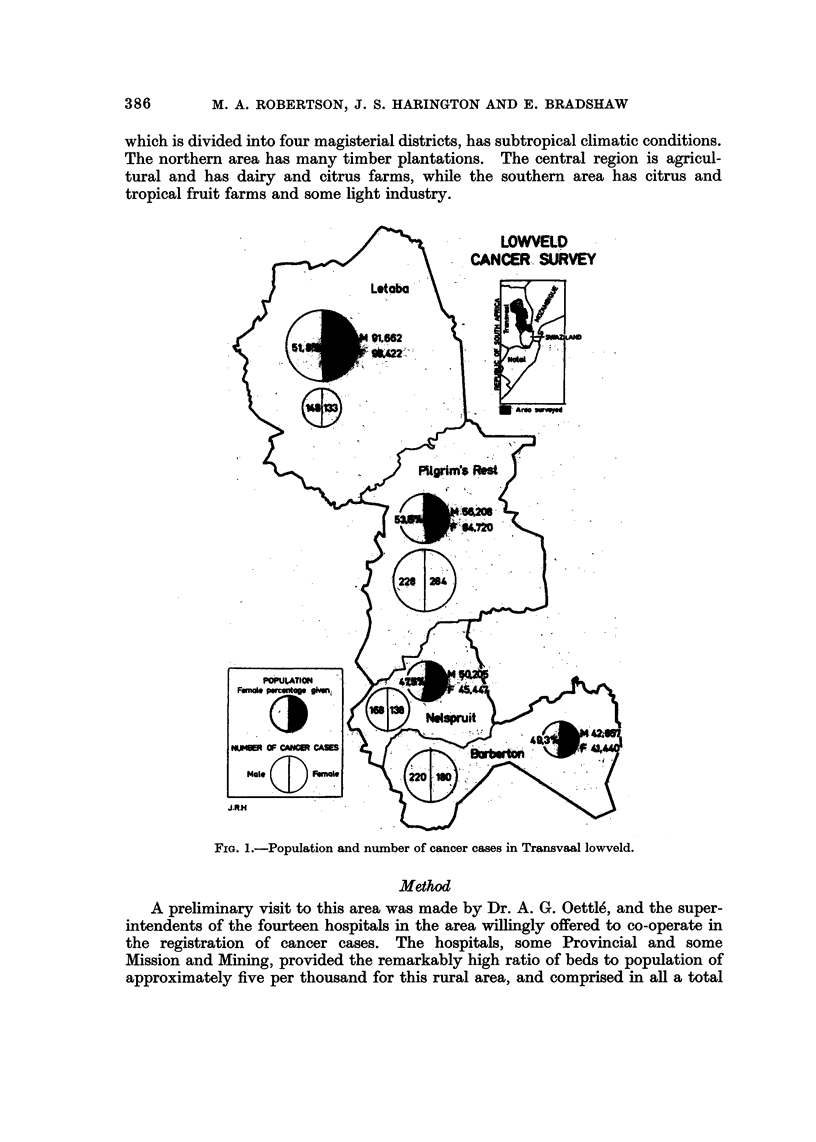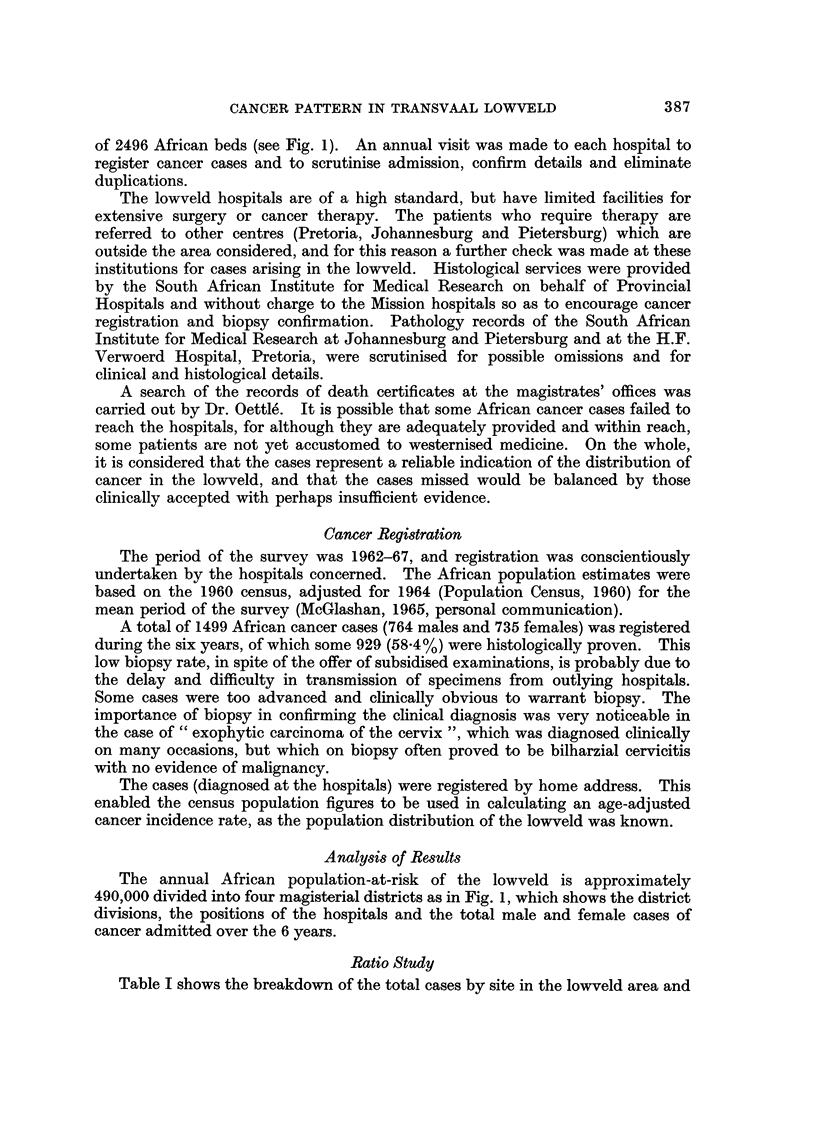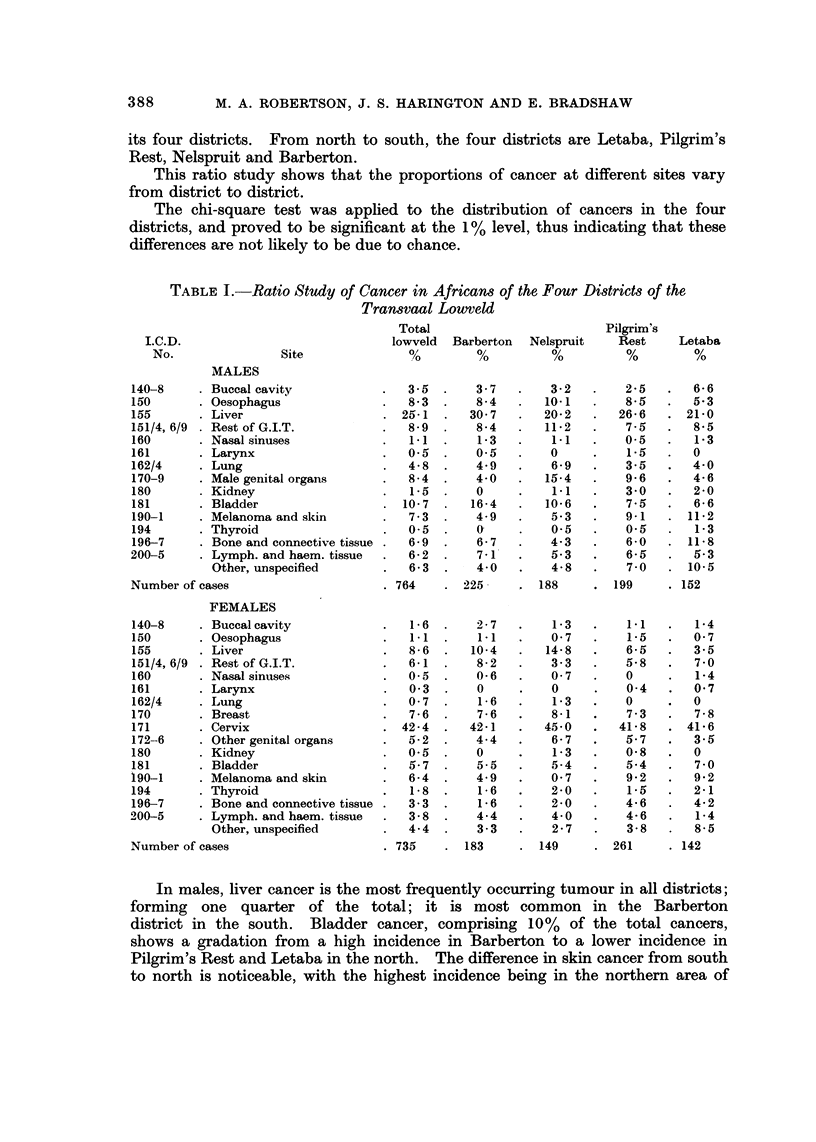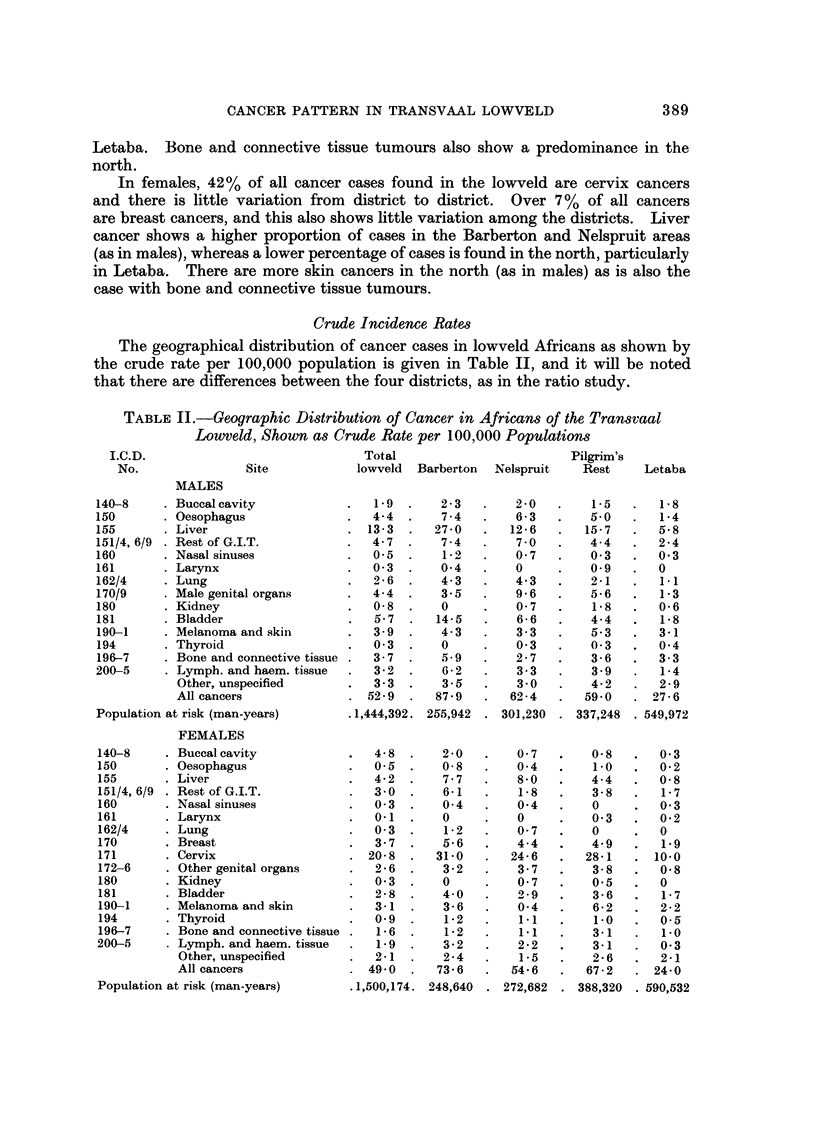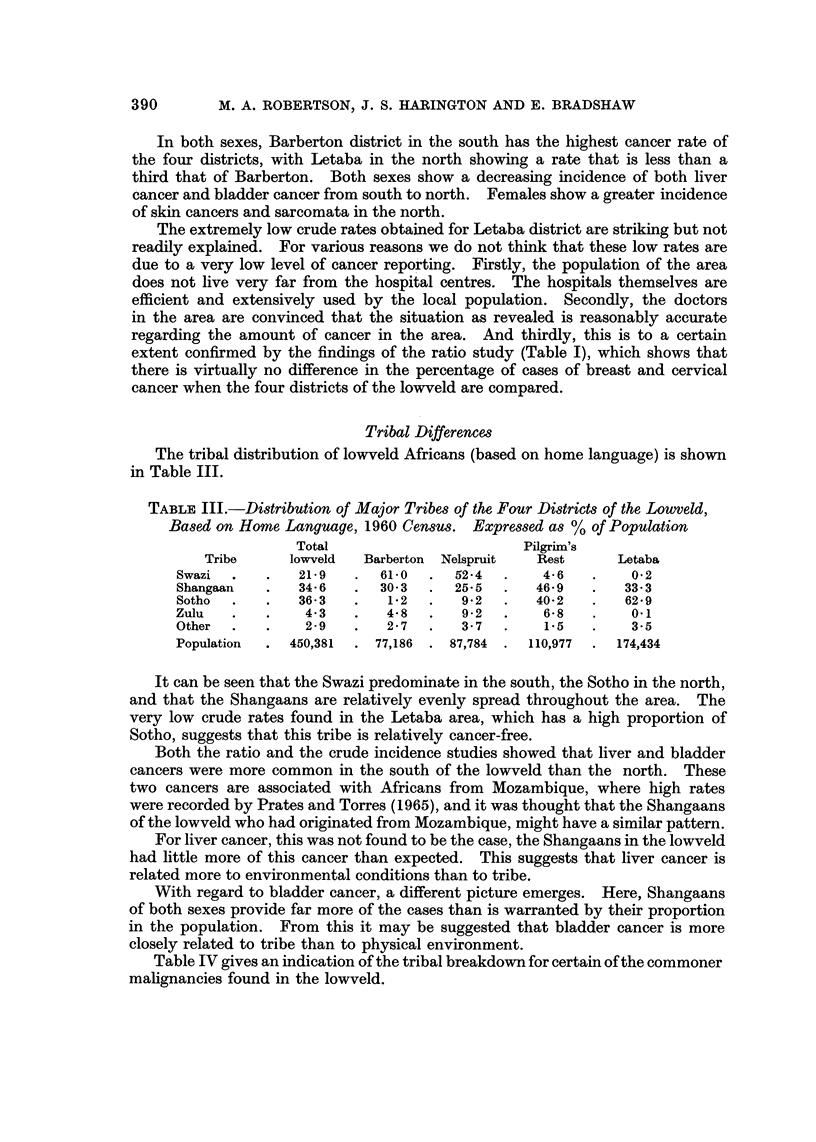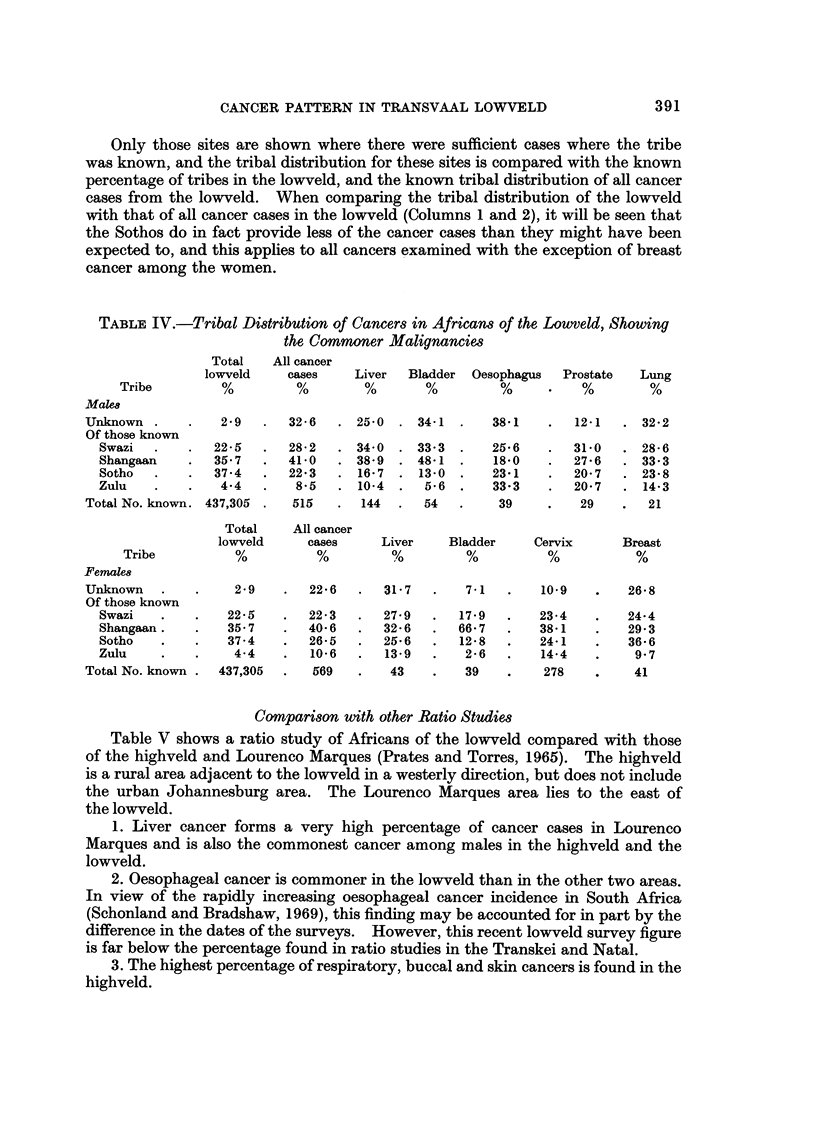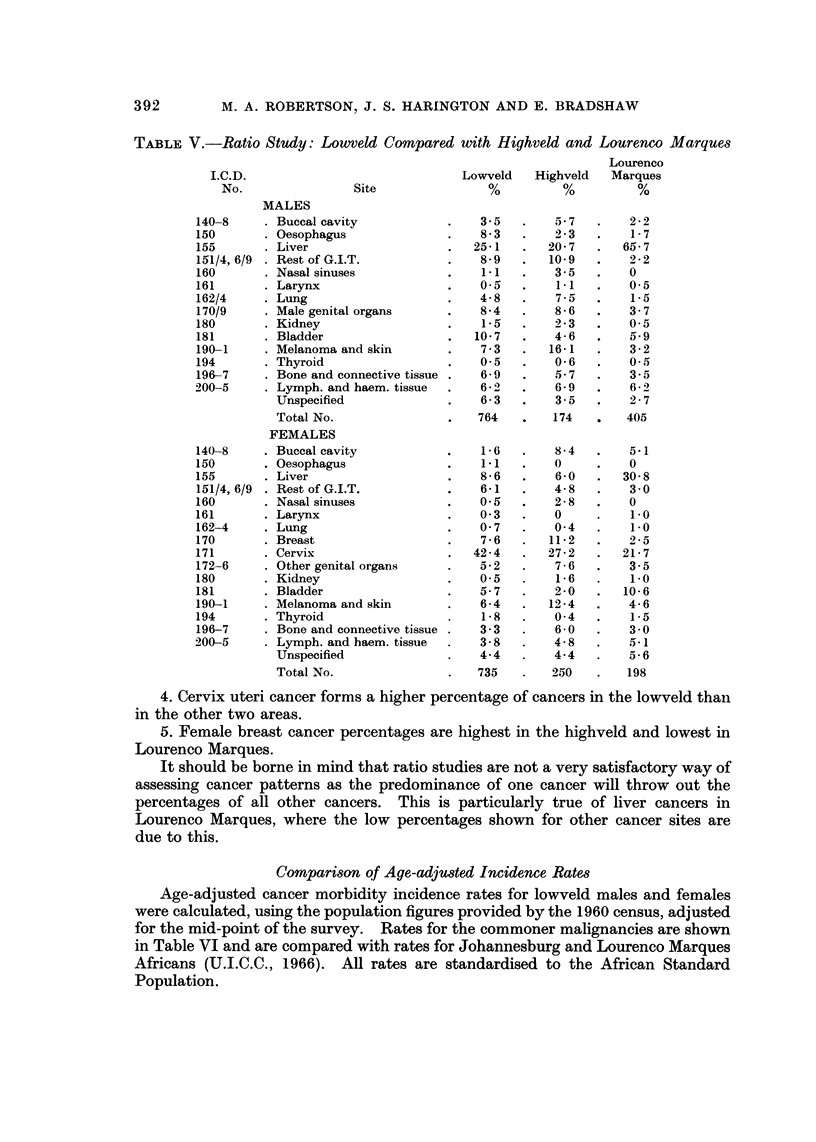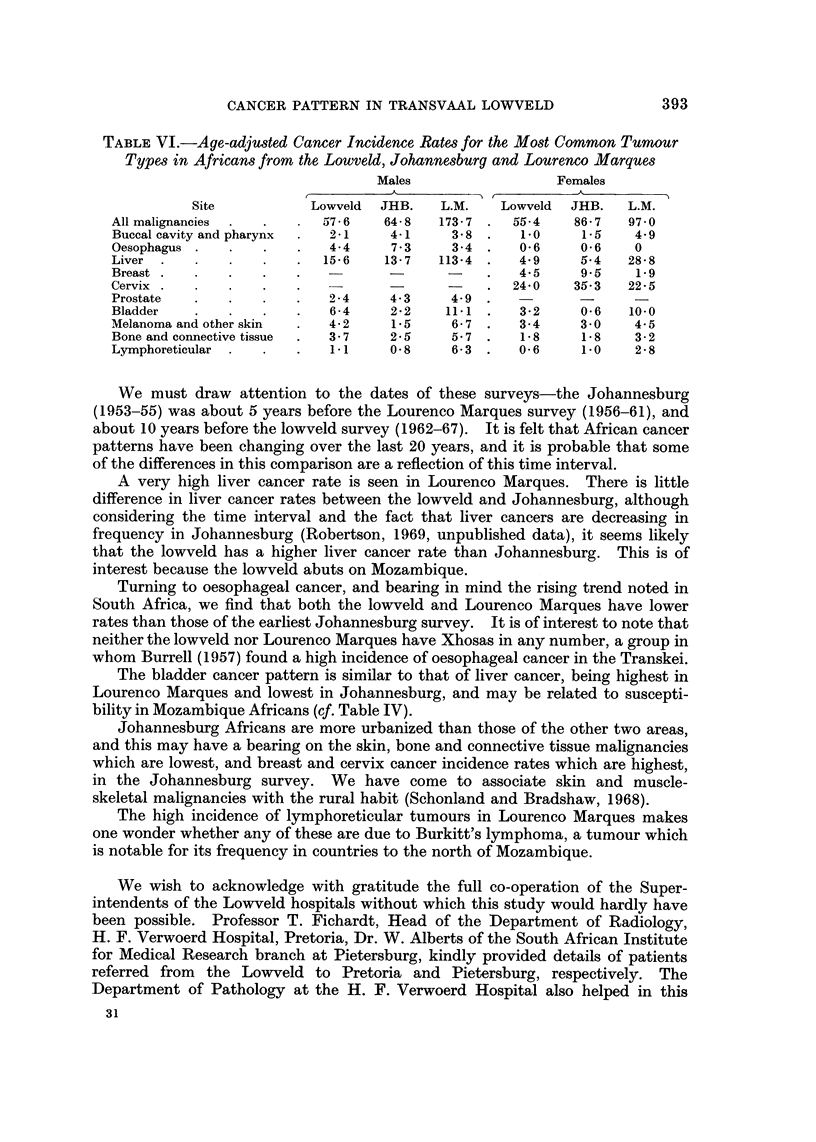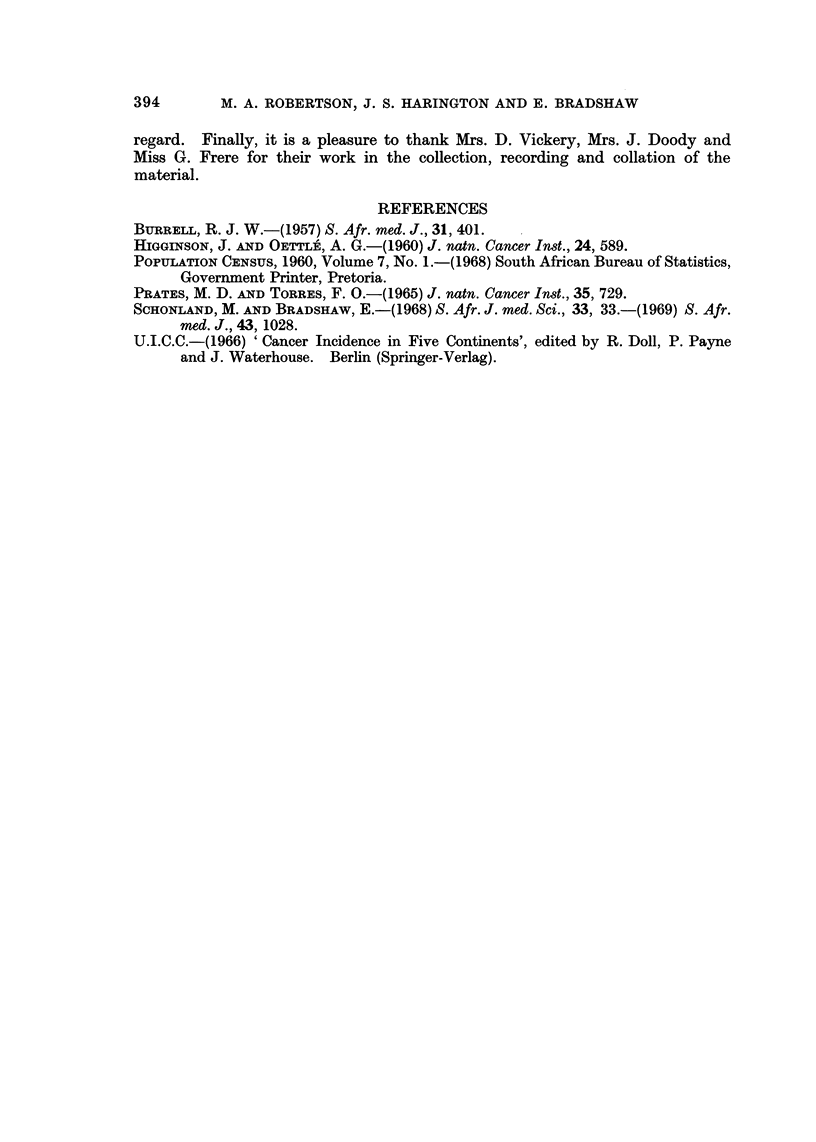# The Cancer Pattern in Africans of the Transvaal Lowveld†

**DOI:** 10.1038/bjc.1971.49

**Published:** 1971-09

**Authors:** M. A. Robertson, J. S. Harington, Evelyn Bradshaw

## Abstract

An attempt has been made to study the Transvaal lowveld by district and tribe in relation to cancer patterns, and to compare these with those of bordering regions.

The lowveld is divided into four districts, running from north to south. There appears to be a real increase in the frequency of liver and bladder cancers from north to south. The low cancer measurements in the most northern district (Letaba) are probably due to low cancer susceptibility. When cancers appear more common in the north, this is of interest seen against the very low cancer rates there. Both skin and musculo-skeletal tumours were commoner in the two northern districts in the ratio study and this was to some extent still true in the crude rate estimations.

Tribally, the Sothos predominate in the north, the Swazis in the south, the Shangaans are evenly distributed through the area. It appears that Sothos are less cancer-susceptible than the other tribes. Looking at geographic and tribal differences together it would seem that liver cancer is related to physical environment whereas bladder cancer is associated with tribe.

An attempt to compare contiguous areas is made in a ratio study comparison between highveld, lowveld and Lourenco Marques. Standardised cancer morbidity incidence rates from three surveys: Lowveld (1962-67), Johannesburg (1953-55), and Lourenco Marques (1956-61) are also compared. Generally speaking, the lowveld occupies a mid-position, both geographically and in terms of cancer patterns, between Lourenco Marques and the highveld—Johannesburg area.


### THE CANCER PATTERN IN AFRICANS OF THE TRANSVAAL LOWVELD†

M. A. ROBERTSON*, J. S. HARINGTON AND EVELYN BRADSHAW

*From the Cancer Research Unit of the National Cancer Association of South Africa, South African Institute for Medical Research, P.O. Box 1038, Johannesburg, South Africa*

Received for publication March 15, 1971

**SUMMARY.—An attempt has been made to study the Transvaal lowveld by district and tribe in relation to cancer patterns, and to compare these with those of bordering regions.**

**The lowveld is divided into four districts, running from north to south. There appears to be a real increase in the frequency of liver and bladder cancers from north to south. The low cancer measurements in the most northern district (Letaba) are probably due to low cancer susceptibility. When cancers appear more common in the north, this is of interest seen against the very low cancer rates there. Both skin and musculo-skeletal tumours were commoner in the two northern districts in the ratio study and this was to some extent still true in the crude rate estimations.**

**Tribally, the Sothos predominate in the north, the Swazis in the south, the Shangaans are evenly distributed through the area. It appears that Sothos are less cancer-susceptible than the other tribes. Looking at geographic and tribal differences together it would seem that liver cancer is related to physical environment whereas bladder cancer is associated with tribe.**

**An attempt to compare contiguous areas is made in a ratio study comparison between highveld, lowveld and Lourenco Marques. Standardised cancer morbidity incidence rates from three surveys: Lowveld (1962–67), Johannesburg (1953–55), and Lourenco Marques (1956–61) are also compared. Generally speaking, the lowveld occupies a mid-position, both geographically and in terms of cancer patterns, between Lourenco Marques and the highveld—Johannesburg area.**

THE Transvaal lowveld area was chosen for a survey of African cancer patterns because of its position between the highveld of the Transvaal, and the coast of Mozambique. Higginson and Oettlé (1960) had reported on cancer patterns of the highveld, while those of Lourenco Marques in Mozambique had been described by Prates and Torres (1965). These surveys varied greatly in the frequency of different types of cancer.

The lowveld represents an area of approximately 15,000 square miles, with a population of some 500,000, which lies roughly some 3000 feet to 500 feet above sea level. It forms a segment gouged out by erosion between the Drakensberg range on the west and the Lebombo range on the east in Mozambique, adjoining the Kruger National Park (see inset map, Fig. 1). The African population is mostly stationary, non-migratory and rural. The major tribes of the areas are Swazi, Sotho and that group known collectively as the Shangaan. The area,

* Deceased, December 4, 1970.
† Reprints from Dr. J. S. Harington.

which is divided into four magisterial districts, has subtropical climatic conditions. The northern area has many timber plantations. The central region is agricultural and has dairy and citrus farms, while the southern area has citrus and tropical fruit farms and some light industry.

FIG. 1.—Population and number of cancer cases in Transvaal lowveld.

### *Method*

A preliminary visit to this area was made by Dr. A. G. Oettlé, and the superintendents of the fourteen hospitals in the area willingly offered to co-operate in the registration of cancer cases. The hospitals, some Provincial and some Mission and Mining, provided the remarkably high ratio of beds to population of approximately five per thousand for this rural area, and comprised in all a total of 2496 African beds (see Fig. 1). An annual visit was made to each hospital to register cancer cases and to scrutinise admission, confirm details and eliminate duplications.

The lowveld hospitals are of a high standard, but have limited facilities for extensive surgery or cancer therapy. The patients who require therapy are referred to other centres (Pretoria, Johannesburg and Pietersburg) which are outside the area considered, and for this reason a further check was made at these institutions for cases arising in the lowveld. Histological services were provided by the South African Institute for Medical Research on behalf of Provincial Hospitals and without charge to the Mission hospitals so as to encourage cancer registration and biopsy confirmation. Pathology records of the South African Institute for Medical Research at Johannesburg and Pietersburg and at the H.F. Verwoerd Hospital, Pretoria, were scrutinised for possible omissions and for clinical and histological details.

A search of the records of death certificates at the magistrates' offices was carried out by Dr. Oettlé. It is possible that some African cancer cases failed to reach the hospitals, for although they are adequately provided and within reach, some patients are not yet accustomed to westernised medicine. On the whole, it is considered that the cases represent a reliable indication of the distribution of cancer in the lowveld, and that the cases missed would be balanced by those clinically accepted with perhaps insufficient evidence.

### *Cancer Registration*

The period of the survey was 1962–67, and registration was conscientiously undertaken by the hospitals concerned. The African population estimates were based on the 1960 census, adjusted for 1964 (Population Census, 1960) for the mean period of the survey (McGlashan, 1965, personal communication).

A total of 1499 African cancer cases (764 males and 735 females) was registered during the six years, of which some 929 (58·4%) were histologically proven. This low biopsy rate, in spite of the offer of subsidised examinations, is probably due to the delay and difficulty in transmission of specimens from outlying hospitals. Some cases were too advanced and clinically obvious to warrant biopsy. The importance of biopsy in confirming the clinical diagnosis was very noticeable in the case of " exophytic carcinoma of the cervix ", which was diagnosed clinically on many occasions, but which on biopsy often proved to be bilharzial cervicitis with no evidence of malignancy.

The cases (diagnosed at the hospitals) were registered by home address. This enabled the census population figures to be used in calculating an age-adjusted cancer incidence rate, as the population distribution of the lowveld was known.

### *Analysis of Results*

The annual African population-at-risk of the lowveld is approximately 490,000 divided into four magisterial districts as in Fig. 1, which shows the district divisions, the positions of the hospitals and the total male and female cases of cancer admitted over the 6 years.

### *Ratio Study*

Table I shows the breakdown of the total cases by site in the lowveld area and its four districts. From north to south, the four districts are Letaba, Pilgrim's Rest, Nelspruit and Barberton.

This ratio study shows that the proportions of cancer at different sites vary from district to district.

The chi-square test was applied to the distribution of cancers in the four districts, and proved to be significant at the 1% level, thus indicating that these differences are not likely to be due to chance.

TABLE I.—*Ratio Study of Cancer in Africans of the Four Districts of the Transvaal Lowveld*

| I.C.D. No. | Site | Total lowveld % | Barberton % | Nelspruit % | Pilgrim's Rest % | Letaba % |
|---|---|---|---|---|---|---|
| | MALES | | | | | |
| 140–8 | Buccal cavity | 3·5 | 3·7 | 3·2 | 2·5 | 6·6 |
| 150 | Oesophagus | 8·3 | 8·4 | 10·1 | 8·5 | 5·3 |
| 155 | Liver | 25·1 | 30·7 | 20·2 | 26·6 | 21·0 |
| 151/4, 6/9 | Rest of G.I.T. | 8·9 | 8·4 | 11·2 | 7·5 | 8·5 |
| 160 | Nasal sinuses | 1·1 | 1·3 | 1·1 | 0·5 | 1·3 |
| 161 | Larynx | 0·5 | 0·5 | 0 | 1·5 | 0 |
| 162/4 | Lung | 4·8 | 4·9 | 6·9 | 3·5 | 4·0 |
| 170–9 | Male genital organs | 8·4 | 4·0 | 15·4 | 9·6 | 4·6 |
| 180 | Kidney | 1·5 | 0 | 1·1 | 3·0 | 2·0 |
| 181 | Bladder | 10·7 | 16·4 | 10·6 | 7·5 | 6·6 |
| 190–1 | Melanoma and skin | 7·3 | 4·9 | 5·3 | 9·1 | 11·2 |
| 194 | Thyroid | 0·5 | 0 | 0·5 | 0·5 | 1·3 |
| 196–7 | Bone and connective tissue | 6·9 | 6·7 | 4·3 | 6·0 | 11·8 |
| 200–5 | Lymph. and haem. tissue | 6·2 | 7·1 | 5·3 | 6·5 | 5·3 |
| | Other, unspecified | 6·3 | 4·0 | 4·8 | 7·0 | 10·5 |
| Number of cases | | 764 | 225 | 188 | 199 | 152 |
| | FEMALES | | | | | |
| 140–8 | Buccal cavity | 1·6 | 2·7 | 1·3 | 1·1 | 1·4 |
| 150 | Oesophagus | 1·1 | 1·1 | 0·7 | 1·5 | 0·7 |
| 155 | Liver | 8·6 | 10·4 | 14·8 | 6·5 | 3·5 |
| 151/4, 6/9 | Rest of G.I.T. | 6·1 | 8·2 | 3·3 | 5·8 | 7·0 |
| 160 | Nasal sinuses | 0·5 | 0·6 | 0·7 | 0 | 1·4 |
| 161 | Larynx | 0·3 | 0 | 0 | 0·4 | 0·7 |
| 162/4 | Lung | 0·7 | 1·6 | 1·3 | 0 | 0 |
| 170 | Breast | 7·6 | 7·6 | 8·1 | 7·3 | 7·8 |
| 171 | Cervix | 42·4 | 42·1 | 45·0 | 41·8 | 41·6 |
| 172–6 | Other genital organs | 5·2 | 4·4 | 6·7 | 5·7 | 3·5 |
| 180 | Kidney | 0·5 | 0 | 1·3 | 0·8 | 0 |
| 181 | Bladder | 5·7 | 5·5 | 5·4 | 5·4 | 7·0 |
| 190–1 | Melanoma and skin | 6·4 | 4·9 | 0·7 | 9·2 | 9·2 |
| 194 | Thyroid | 1·8 | 1·6 | 2·0 | 1·5 | 2·1 |
| 196–7 | Bone and connective tissue | 3·3 | 1·6 | 2·0 | 4·6 | 4·2 |
| 200–5 | Lymph. and haem. tissue | 3·8 | 4·4 | 4·0 | 4·6 | 1·4 |
| | Other, unspecified | 4·4 | 3·3 | 2·7 | 3·8 | 8·5 |
| Number of cases | | 735 | 183 | 149 | 261 | 142 |

In males, liver cancer is the most frequently occurring tumour in all districts; forming one quarter of the total; it is most common in the Barberton district in the south. Bladder cancer, comprising 10% of the total cancers, shows a gradation from a high incidence in Barberton to a lower incidence in Pilgrim's Rest and Letaba in the north. The difference in skin cancer from south to north is noticeable, with the highest incidence being in the northern area of

Letaba. Bone and connective tissue tumours also show a predominance in the north.

In females, 42% of all cancer cases found in the lowveld are cervix cancers and there is little variation from district to district. Over 7% of all cancers are breast cancers, and this also shows little variation among the districts. Liver cancer shows a higher proportion of cases in the Barberton and Nelspruit areas (as in males), whereas a lower percentage of cases is found in the north, particularly in Letaba. There are more skin cancers in the north (as in males) as is also the case with bone and connective tissue tumours.

### *Crude Incidence Rates*

The geographical distribution of cancer cases in lowveld Africans as shown by the crude rate per 100,000 population is given in Table II, and it will be noted that there are differences between the four districts, as in the ratio study.

TABLE II.—*Geographic Distribution of Cancer in Africans of the Transvaal Lowveld, Shown as Crude Rate per* 100,000 *Populations*

| I.C.D. No. | Site | Total lowveld | Barberton | Nelspruit | Pilgrim's Rest | Letaba |
|---|---|---|---|---|---|---|
| | MALES | | | | | |
| 140–8 | Buccal cavity | 1·9 | 2·3 | 2·0 | 1·5 | 1·8 |
| 150 | Oesophagus | 4·4 | 7·4 | 6·3 | 5·0 | 1·4 |
| 155 | Liver | 13·3 | 27·0 | 12·6 | 15·7 | 5·8 |
| 151/4, 6/9 | Rest of G.I.T. | 4·7 | 7·4 | 7·0 | 4·4 | 2·4 |
| 160 | Nasal sinuses | 0·5 | 1·2 | 0·7 | 0·3 | 0·3 |
| 161 | Larynx | 0·3 | 0·4 | 0 | 0·9 | 0 |
| 162/4 | Lung | 2·6 | 4·3 | 4·3 | 2·1 | 1·1 |
| 170/9 | Male genital organs | 4·4 | 3·5 | 9·6 | 5·6 | 1·3 |
| 180 | Kidney | 0·8 | 0 | 0·7 | 1·8 | 0·6 |
| 181 | Bladder | 5·7 | 14·5 | 6·6 | 4·4 | 1·8 |
| 190–1 | Melanoma and skin | 3·9 | 4·3 | 3·3 | 5·3 | 3·1 |
| 194 | Thyroid | 0·3 | 0 | 0·3 | 0·3 | 0·4 |
| 196–7 | Bone and connective tissue | 3·7 | 5·9 | 2·7 | 3·6 | 3·3 |
| 200–5 | Lymph. and haem. tissue | 3·2 | 6·2 | 3·3 | 3·9 | 1·4 |
| | Other, unspecified | 3·3 | 3·5 | 3·0 | 4·2 | 2·9 |
| | All cancers | 52·9 | 87·9 | 62·4 | 59·0 | 27·6 |
| Population at risk (man-years) | | 1,444,392 | 255,942 | 301,230 | 337,248 | 549,972 |
| | FEMALES | | | | | |
| 140–8 | Buccal cavity | 4·8 | 2·0 | 0·7 | 0·8 | 0·3 |
| 150 | Oesophagus | 0·5 | 0·8 | 0·4 | 1·0 | 0·2 |
| 155 | Liver | 4·2 | 7·7 | 8·0 | 4·4 | 0·8 |
| 151/4, 6/9 | Rest of G.I.T. | 3·0 | 6·1 | 1·8 | 3·8 | 1·7 |
| 160 | Nasal sinuses | 0·3 | 0·4 | 0·4 | 0 | 0·3 |
| 161 | Larynx | 0·1 | 0 | 0 | 0·3 | 0·2 |
| 162/4 | Lung | 0·3 | 1·2 | 0·7 | 0 | 0 |
| 170 | Breast | 3·7 | 5·6 | 4·4 | 4·9 | 1·9 |
| 171 | Cervix | 20·8 | 31·0 | 24·6 | 28·1 | 10·0 |
| 172–6 | Other genital organs | 2·6 | 3·2 | 3·7 | 3·8 | 0·8 |
| 180 | Kidney | 0·3 | 0 | 0·7 | 0·5 | 0 |
| 181 | Bladder | 2·8 | 4·0 | 2·9 | 3·6 | 1·7 |
| 190–1 | Melanoma and skin | 3·1 | 3·6 | 0·4 | 6·2 | 2·2 |
| 194 | Thyroid | 0·9 | 1·2 | 1·1 | 1·0 | 0·5 |
| 196–7 | Bone and connective tissue | 1·6 | 1·2 | 1·1 | 3·1 | 1·0 |
| 200–5 | Lymph. and haem. tissue | 1·9 | 3·2 | 2·2 | 3·1 | 0·3 |
| | Other, unspecified | 2·1 | 2·4 | 1·5 | 2·6 | 2·1 |
| | All cancers | 49·0 | 73·6 | 54·6 | 67·2 | 24·0 |
| Population at risk (man-years) | | 1,500,174 | 248,640 | 272,682 | 388,320 | 590,532 |

In both sexes, Barberton district in the south has the highest cancer rate of the four districts, with Letaba in the north showing a rate that is less than a third that of Barberton. Both sexes show a decreasing incidence of both liver cancer and bladder cancer from south to north. Females show a greater incidence of skin cancers and sarcomata in the north.

The extremely low crude rates obtained for Letaba district are striking but not readily explained. For various reasons we do not think that these low rates are due to a very low level of cancer reporting. Firstly, the population of the area does not live very far from the hospital centres. The hospitals themselves are efficient and extensively used by the local population. Secondly, the doctors in the area are convinced that the situation as revealed is reasonably accurate regarding the amount of cancer in the area. And thirdly, this is to a certain extent confirmed by the findings of the ratio study (Table I), which shows that there is virtually no difference in the percentage of cases of breast and cervical cancer when the four districts of the lowveld are compared.

### *Tribal Differences*

The tribal distribution of lowveld Africans (based on home language) is shown in Table III.

TABLE III.—*Distribution of Major Tribes of the Four Districts of the Lowveld, Based on Home Language,* 1960 *Census. Expressed as % of Population*

| Tribe | Total lowveld | Barberton | Nelspruit | Pilgrim's Rest | Letaba |
|---|---|---|---|---|---|
| Swazi | 21·9 | 61·0 | 52·4 | 4·6 | 0·2 |
| Shangaan | 34·6 | 30·3 | 25·5 | 46·9 | 33·3 |
| Sotho | 36·3 | 1·2 | 9·2 | 40·2 | 62·9 |
| Zulu | 4·3 | 4·8 | 9·2 | 6·8 | 0·1 |
| Other | 2·9 | 2·7 | 3·7 | 1·5 | 3·5 |
| Population | 450,381 | 77,186 | 87,784 | 110,977 | 174,434 |

It can be seen that the Swazi predominate in the south, the Sotho in the north, and that the Shangaans are relatively evenly spread throughout the area. The very low crude rates found in the Letaba area, which has a high proportion of Sotho, suggests that this tribe is relatively cancer-free.

Both the ratio and the crude incidence studies showed that liver and bladder cancers were more common in the south of the lowveld than the north. These two cancers are associated with Africans from Mozambique, where high rates were recorded by Prates and Torres (1965), and it was thought that the Shangaans of the lowveld who had originated from Mozambique, might have a similar pattern.

For liver cancer, this was not found to be the case, the Shangaans in the lowveld had little more of this cancer than expected. This suggests that liver cancer is related more to environmental conditions than to tribe.

With regard to bladder cancer, a different picture emerges. Here, Shangaans of both sexes provide far more of the cases than is warranted by their proportion in the population. From this it may be suggested that bladder cancer is more closely related to tribe than to physical environment.

Table IV gives an indication of the tribal breakdown for certain of the commoner malignancies found in the lowveld.

Only those sites are shown where there were sufficient cases where the tribe was known, and the tribal distribution for these sites is compared with the known percentage of tribes in the lowveld, and the known tribal distribution of all cancer cases from the lowveld. When comparing the tribal distribution of the lowveld with that of all cancer cases in the lowveld (Columns 1 and 2), it will be seen that the Sothos do in fact provide less of the cancer cases than they might have been expected to, and this applies to all cancers examined with the exception of breast cancer among the women.

TABLE IV.—*Tribal Distribution of Cancers in Africans of the Lowveld, Showing the Commoner Malignancies*

| Tribe | Total lowveld % | All cancer cases % | Liver % | Bladder % | Oesophagus % | Prostate % | Lung % |
|---|---|---|---|---|---|---|---|
| *Males* | | | | | | | |
| Unknown | 2·9 | 32·6 | 25·0 | 34·1 | 38·1 | 12·1 | 32·2 |
| Of those known | | | | | | | |
| Swazi | 22·5 | 28·2 | 34·0 | 33·3 | 25·6 | 31·0 | 28·6 |
| Shangaan | 35·7 | 41·0 | 38·9 | 48·1 | 18·0 | 27·6 | 33·3 |
| Sotho | 37·4 | 22·3 | 16·7 | 13·0 | 23·1 | 20·7 | 23·8 |
| Zulu | 4·4 | 8·5 | 10·4 | 5·6 | 33·3 | 20·7 | 14·3 |
| Total No. known | 437,305 | 515 | 144 | 54 | 39 | 29 | 21 |

| Tribe | Total lowveld % | All cancer cases % | Liver % | Bladder % | Cervix % | Breast % |
|---|---|---|---|---|---|---|
| *Females* | | | | | | |
| Unknown | 2·9 | 22·6 | 31·7 | 7·1 | 10·9 | 26·8 |
| Of those known | | | | | | |
| Swazi | 22·5 | 22·3 | 27·9 | 17·9 | 23·4 | 24·4 |
| Shangaan | 35·7 | 40·6 | 32·6 | 66·7 | 38·1 | 29·3 |
| Sotho | 37·4 | 26·5 | 25·6 | 12·8 | 24·1 | 36·6 |
| Zulu | 4·4 | 10·6 | 13·9 | 2·6 | 14·4 | 9·7 |
| Total No. known | 437,305 | 569 | 43 | 39 | 278 | 41 |

*Comparison with other Ratio Studies*

Table V shows a ratio study of Africans of the lowveld compared with those of the highveld and Lourenco Marques (Prates and Torres, 1965). The highveld is a rural area adjacent to the lowveld in a westerly direction, but does not include the urban Johannesburg area. The Lourenco Marques area lies to the east of the lowveld.

1. Liver cancer forms a very high percentage of cancer cases in Lourenco Marques and is also the commonest cancer among males in the highveld and the lowveld.

2. Oesophageal cancer is commoner in the lowveld than in the other two areas. In view of the rapidly increasing oesophageal cancer incidence in South Africa (Schonland and Bradshaw, 1969), this finding may be accounted for in part by the difference in the dates of the surveys. However, this recent lowveld survey figure is far below the percentage found in ratio studies in the Transkei and Natal.

3. The highest percentage of respiratory, buccal and skin cancers is found in the highveld.

TABLE V.—*Ratio Study: Lowveld Compared with Highveld and Lourenco Marques*

| I.C.D. No. | Site | Lowveld % | Highveld % | Lourenco Marques % |
|---|---|---|---|---|
| | MALES | | | |
| 140–8 | Buccal cavity | 3·5 | 5·7 | 2·2 |
| 150 | Oesophagus | 8·3 | 2·3 | 1·7 |
| 155 | Liver | 25·1 | 20·7 | 65·7 |
| 151/4, 6/9 | Rest of G.I.T. | 8·9 | 10·9 | 2·2 |
| 160 | Nasal sinuses | 1·1 | 3·5 | 0 |
| 161 | Larynx | 0·5 | 1·1 | 0·5 |
| 162/4 | Lung | 4·8 | 7·5 | 1·5 |
| 170/9 | Male genital organs | 8·4 | 8·6 | 3·7 |
| 180 | Kidney | 1·5 | 2·3 | 0·5 |
| 181 | Bladder | 10·7 | 4·6 | 5·9 |
| 190–1 | Melanoma and skin | 7·3 | 16·1 | 3·2 |
| 194 | Thyroid | 0·5 | 0·6 | 0·5 |
| 196–7 | Bone and connective tissue | 6·9 | 5·7 | 3·5 |
| 200–5 | Lymph. and haem. tissue | 6·2 | 6·9 | 6·2 |
| | Unspecified | 6·3 | 3·5 | 2·7 |
| | Total No. | 764 | 174 | 405 |
| | FEMALES | | | |
| 140–8 | Buccal cavity | 1·6 | 8·4 | 5·1 |
| 150 | Oesophagus | 1·1 | 0 | 0 |
| 155 | Liver | 8·6 | 6·0 | 30·8 |
| 151/4, 6/9 | Rest of G.I.T. | 6·1 | 4·8 | 3·0 |
| 160 | Nasal sinuses | 0·5 | 2·8 | 0 |
| 161 | Larynx | 0·3 | 0 | 1·0 |
| 162–4 | Lung | 0·7 | 0·4 | 1·0 |
| 170 | Breast | 7·6 | 11·2 | 2·5 |
| 171 | Cervix | 42·4 | 27·2 | 21·7 |
| 172–6 | Other genital organs | 5·2 | 7·6 | 3·5 |
| 180 | Kidney | 0·5 | 1·6 | 1·0 |
| 181 | Bladder | 5·7 | 2·0 | 10·6 |
| 190–1 | Melanoma and skin | 6·4 | 12·4 | 4·6 |
| 194 | Thyroid | 1·8 | 0·4 | 1·5 |
| 196–7 | Bone and connective tissue | 3·3 | 6·0 | 3·0 |
| 200–5 | Lymph. and haem. tissue | 3·8 | 4·8 | 5·1 |
| | Unspecified | 4·4 | 4·4 | 5·6 |
| | Total No. | 735 | 250 | 198 |

4. Cervix uteri cancer forms a higher percentage of cancers in the lowveld than in the other two areas.

5. Female breast cancer percentages are highest in the highveld and lowest in Lourenco Marques.

It should be borne in mind that ratio studies are not a very satisfactory way of assessing cancer patterns as the predominance of one cancer will throw out the percentages of all other cancers. This is particularly true of liver cancers in Lourenco Marques, where the low percentages shown for other cancer sites are due to this.

### *Comparison of Age-adjusted Incidence Rates*

Age-adjusted cancer morbidity incidence rates for lowveld males and females were calculated, using the population figures provided by the 1960 census, adjusted for the mid-point of the survey. Rates for the commoner malignancies are shown in Table VI and are compared with rates for Johannesburg and Lourenco Marques Africans (U.I.C.C., 1966). All rates are standardised to the African Standard Population.

TABLE VI.—*Age-adjusted Cancer Incidence Rates for the Most Common Tumour Types in Africans from the Lowveld, Johannesburg and Lourenco Marques*

| | Males | | | Females | | |
|---|---|---|---|---|---|---|
| Site | Lowveld | JHB. | L.M. | Lowveld | JHB. | L.M. |
| All malignancies | 57·6 | 64·8 | 173·7 | 55·4 | 86·7 | 97·0 |
| Buccal cavity and pharynx | 2·1 | 4·1 | 3·8 | 1·0 | 1·5 | 4·9 |
| Oesophagus | 4·4 | 7·3 | 3·4 | 0·6 | 0·6 | 0 |
| Liver | 15·6 | 13·7 | 113·4 | 4·9 | 5·4 | 28·8 |
| Breast | — | — | — | 4·5 | 9·5 | 1·9 |
| Cervix | — | — | — | 24·0 | 35·3 | 22·5 |
| Prostate | 2·4 | 4·3 | 4·9 | — | — | — |
| Bladder | 6·4 | 2·2 | 11·1 | 3·2 | 0·6 | 10·0 |
| Melanoma and other skin | 4·2 | 1·5 | 6·7 | 3·4 | 3·0 | 4·5 |
| Bone and connective tissue | 3·7 | 2·5 | 5·7 | 1·8 | 1·8 | 3·2 |
| Lymphoreticular | 1·1 | 0·8 | 6·3 | 0·6 | 1·0 | 2·8 |

We must draw attention to the dates of these surveys—the Johannesburg (1953–55) was about 5 years before the Lourenco Marques survey (1956–61), and about 10 years before the lowveld survey (1962–67). It is felt that African cancer patterns have been changing over the last 20 years, and it is probable that some of the differences in this comparison are a reflection of this time interval.

A very high liver cancer rate is seen in Lourenco Marques. There is little difference in liver cancer rates between the lowveld and Johannesburg, although considering the time interval and the fact that liver cancers are decreasing in frequency in Johannesburg (Robertson, 1969, unpublished data), it seems likely that the lowveld has a higher liver cancer rate than Johannesburg. This is of interest because the lowveld abuts on Mozambique.

Turning to oesophageal cancer, and bearing in mind the rising trend noted in South Africa, we find that both the lowveld and Lourenco Marques have lower rates than those of the earliest Johannesburg survey. It is of interest to note that neither the lowveld nor Lourenco Marques have Xhosas in any number, a group in whom Burrell (1957) found a high incidence of oesophageal cancer in the Transkei.

The bladder cancer pattern is similar to that of liver cancer, being highest in Lourenco Marques and lowest in Johannesburg, and may be related to susceptibility in Mozambique Africans (*cf.* Table IV).

Johannesburg Africans are more urbanized than those of the other two areas, and this may have a bearing on the skin, bone and connective tissue malignancies which are lowest, and breast and cervix cancer incidence rates which are highest, in the Johannesburg survey. We have come to associate skin and muscle-skeletal malignancies with the rural habit (Schonland and Bradshaw, 1968).

The high incidence of lymphoreticular tumours in Lourenco Marques makes one wonder whether any of these are due to Burkitt's lymphoma, a tumour which is notable for its frequency in countries to the north of Mozambique.

We wish to acknowledge with gratitude the full co-operation of the Superintendents of the Lowveld hospitals without which this study would hardly have been possible. Professor T. Fichardt, Head of the Department of Radiology, H. F. Verwoerd Hospital, Pretoria, Dr. W. Alberts of the South African Institute for Medical Research branch at Pietersburg, kindly provided details of patients referred from the Lowveld to Pretoria and Pietersburg, respectively. The Department of Pathology at the H. F. Verwoerd Hospital also helped in this regard. Finally, it is a pleasure to thank Mrs. D. Vickery, Mrs. J. Doody and Miss G. Frere for their work in the collection, recording and collation of the material.

### REFERENCES

Burrell, R. J. W.—(1957) *S. Afr. med. J.*, **31**, 401.
Higginson, J. and Oettlé, A. G.—(1960) *J. natn. Cancer Inst.*, **24**, 589.
Population Census, 1960, Volume 7, No. 1.—(1968) South African Bureau of Statistics, Government Printer, Pretoria.
Prates, M. D. and Torres, F. O.—(1965) *J. natn. Cancer Inst.*, **35**, 729.
Schonland, M. and Bradshaw, E.—(1968) *S. Afr. J. med. Sci.*, **33**, 33.—(1969) *S. Afr. med. J.*, **43**, 1028.
U.I.C.C.—(1966) ‘ Cancer Incidence in Five Continents’, edited by R. Doll, P. Payne and J. Waterhouse. Berlin (Springer-Verlag).